# Central corneal regularization (CCR): an alternative approach in keratoconus treatment

**DOI:** 10.1186/s40662-019-0165-y

**Published:** 2019-12-16

**Authors:** Giulio Mulè, Shihao Chen, Jia Zhang, Wen Zhou, Vasileios Selimis, Aleksandar Stojanovic, Ioannis M. Aslanides

**Affiliations:** 1 iVis Trento Center, San Camillo Hospital, Trento, Italy; 2 Eye Hospital, Wenzhou Medical University, Wenzhou, China; 3 SynsLaser Kirurgi AS, Skippergata 7, Tromso, Norway; 4 Emmetropia Mediterranean Eye Institute, Plateia Eleftherias 44, Heraklion, 71201 Crete, Greece

**Keywords:** Central corneal regularization, Corneal collagen cross-linking, Transepithelial, Excimer laser, Keratoconus

## Abstract

**Background:**

To evaluate the safety and efficacy of an approach that combines corneal customized transepithelial therapeutic ablation to treat irregular corneal optics and accelerated corneal collagen cross-linking (CXL) to strengthen the corneal tissue and stop the progression of keratoconus. The transepithelial therapeutic ablation applied a novel concept named central corneal regularization (CCR) which could correct the corneal morphological irregularities and the eye’s spherocylindrical refractive error with minimal stromal tissue removal.

**Methods:**

Retrospective study. Eyes that underwent CCR combined with CXL were evaluated preoperatively and up to 12 months postoperatively for visual acuity, subjective refraction, corneal haze, pachymetry and maximum keratometry (Kmax).

**Results:**

Twenty four eyes of 24 patients with a mean age of 28.92 ± 9.88 years were treated. The mean spherical equivalent (SE) refractive error changed from − 0.74 ± 1.17 D preoperatively to − 1.05 ± 1.52 D at 12 months postoperatively. The mean uncorrected distance visual acuity (UDVA) and corrected distance visual acuity (CDVA) improved. No eye lost lines of CDVA, 21 had a mean improvement of 3.21 lines. The mean cylinder error and Kmax value dropped from − 3.06 ± 1.83 D and 51.38 ± 3.29 D to − 1.04 ± 0.80 D and 48.70 ± 2.58 D, respectively. The mean haze score at 3, 6 and 12 months was 0.56, 0.19 and 0.06, respectively.

**Conclusions:**

CCR combined with CXL offers promising results as a safe and effective treatment in keratoconic patients.

## Background

Keratoconus is a disease in which a local failure of the corneal biomechanical strength results in abnormal corneal protrusion and thinning, leading to poor visual acuity and quality [1, 2]. Several approaches have been evaluated for correction of vision or for slowing down the progression of keratoconus. In addition to corneal transplantation, normally kept as the last resource, specialists’ common practice includes the use of rigid contact lenses, implantation of intrastromal ring segments and, in the last decade, corneal cross-linking (CXL). The goal of the last-mentioned approach has been to mostly stabilize the progression of the keratoectatic process, without specifically addressing or controlling vision [1, 3].

Previous studies concerning CXL alone demonstrated postoperative stability or small improvements in vision. In their > 3 years follow-up retrospective study, Raiskup-Wolf et al. [4], demonstrated an improvement in corrected distance visual acuity (CDVA) of 0.15 logMAR while Wittig-Silva et al. [5] in their prospective comparative study with > 3 years follow-up demonstrated an improvement of 0.09 logMAR in CDVA and of 0.15 logMAR in uncorrected distance visual acuity (UDVA).

In order to gain a better control of visual outcomes in addition to strengthening the cornea, CXL has been combined with other procedures such as laser vision correction, implantation of corneal ring segments, or use of phakic or aphakic intraocular lenses, thus improving eye optics. It seems that corneal surface ablation, performed in the same session as CXL or sequentially, has been the most popular of those procedures [1, 6, 7]. The combined treatment consisting of CXL preceded by a minimally invasive customized trans-epithelial corneal regularization seems to be a promising approach to freezing the keratoectatic process in an optically regularized cornea and achieving a potentially stable improvement of the quality of vision.

An innovative concept introduced by the iVis Suite customized excimer laser ablation treatment platform (iVis Technologies S. r. l., Taranto, Italy), named Central Corneal Regularization (CCR), was reported for the use in keratoconic corneas [6, 7]. The system uses topo/tomography-guided ablation to regularize the corneal shape by eliminating the higher order corneal morphological aberrations (HOCMAs) within a very narrow central optical zone, while the treatment of the HOCMAs towards the periphery gradually decreases within a large total ablation diameter. With this approach, the stromal tissue removal and the surgical invasiveness is kept to a minimum, while the customized “connecting refraction zone” instead of the traditional transition zone keeps the continuity of the corneal refractive power, with an aim of reducing the glare and haloes as well as the risk of regression. The current study retrospectively evaluates the clinical outcomes using the outlined approach combined with CXL performed right after the CCR, within the same surgical session.

## Methods

This pilot study comprises 24 eyes (13 right and 11 left eyes) of retrospectively recruited 24 patients with keratoconus who underwent combined CCR and CXL, in a private eye clinic in Crete, Greece. Mean age was 28.92 years (SD 9.88, range 14 to 53).

Inclusion criteria comprised clinically diagnosed keratoconus with topographic changes consistent with moderate severity (Amsler-Krumeich stage I-II). All patients had visual acuity affected to the point where spectacles could not offer satisfactory results. Exclusion criteria consisted the presence of significant scars on the cornea, presence of other corneal disease, inflammation, or other disorders that could affect the final outcomes. The research was conducted according to the principles of the Declaration of Helsinki and ethical approval was granted by the institute’s review board.

Pre-operative assessment included uncorrected (UDVA) and corrected (CDVA) distant visual acuity, cycloplegic and non-cycloplegic manifest refraction, and slit lamp examination, including dilated fundoscopy and measurement of intraocular pressure. Scheimpflug-based Imaging corneal topo/tomography, obtained by Precisio (iVis Technologies S. r. l., Taranto, Italy), was used for a preoperative diagnostic as the basis for custom surgery planning and pre- and postoperative structural and optical analysis, including the evaluation of HOCMAs [6, 8]. For the measurement of the latter, a parameter describing optical regularity of the corneal surface, named HOCMAs, was introduced by the manufacturer of the Precisio. The HOCMAs are cumulatively all the refractive aberrations above the second order i.e., sphere and cylinder.

**Fig. 1:**
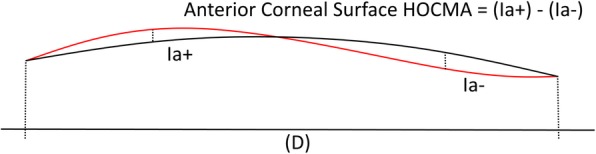
Higher order corneal morphological aberrations (HOCMAs) calculation. HOCMAs – Morphological substrate for the anterior corneal surface optical aberrations above the second order, calculated with respect to the best fit toric surface within a predefined diameter. The HOCMAs of the anterior corneal surface are calculated as the difference between the positive index of irregularity of the anterior corneal surface (Ia+) and the negative index of irregularity of the anterior corneal surface (Ia-), which are respectively the maximum and the minimum difference between the anterior corneal surface and the best fit toric surface calculated within the predefined domain (D)

The measurement of the HOCMAs provides an indication of the regularity of surface, which translates to the quality of vision of the patient: the higher the measured HOCMAs, the worse the patient quality of vision. The HOCMAs are clinically induced either secondary to complicated refractive, or other eye surgery, corneal injuries or post-keratitis scarring in stable corneas, or in unstable corneas due to ectatic disease. The HOCMAs of the anterior corneal surface are calculated as the difference between the positive index of irregularity of the anterior corneal surface (Ia+) and the negative index of irregularity of the anterior corneal surface (Ia-), which are respectively the maximum and the minimum difference between the anterior corneal surface and the best fit toric surface calculated within the predefined domain (D), see enclosed picture (Fig. 1).

### Surgical technique

All treatments were planned using the Corneal Interactive Programmed Topographic Ablation (CIPTA) software (iVis Technologies s. r. l., Taranto, Italy) in CCR mode [8, 9]. High definition anterior and posterior corneal topography and pachymetry acquired by the Precisio were imported to CIPTA to design an ablation that aims to transform the preoperative irregular corneal morphology into a regular aconic shape of desired curvature, defined as the expected post-operative anterior corneal curvature according to the programmed treatment, and correct the corneal morphological irregularities and the eye’s spherocylindrical refractive error. To achieve a minimal tissue removal from the biomechanically compromised cornea, a full regularization is aimed only within a narrow optical zone (1.0–1.5 mm in diameter), while the quality of the postoperative corneal optics is addressed by gradually fading custom ablation effect towards the periphery, within a total ablation zone of up to 9.8 mm in diameter. The large “connecting refractive zone” between the central optical zone and the untreated periphery features a smooth customized transition with a constant radial slope. The connecting zone is the surface between the refractive zone and the untouched corneal surface, designed with a constant slope in each radial direction, resulting in linear increase or decrease of curvature. All the treatments were planned to leave at least a 400 μm residual stromal bed.

Transepithelial single-step laser treatment, comprising a predefined ablation profile to achieve the epithelial removal and a customized component to achieve the corneal regularization, was executed with an uninterrupted ablation [7]. The predefined ablation profile to remove the epithelium was preprogrammed with a proprietary algorithm. The excimer laser used for the customized transepithelial treatment was the iRES (iVis Technologies S. r. l., Taranto, Italy), that has a small spot size of 0.65 mm and a frequency of up to 1000 Hz, delivered on the cornea. The laser employs a patented concept of constant frequency per area in order to prevent overheating due to its high frequency.

Prior to ablation, a semi-moist Merocell sponge (Medtronic Inc., USA) dipped in balanced salt solution (BSS) was applied on the corneal surface to avoid uneven wetting [1, 10]. After the laser ablation, the cornea was cooled with chilled BSS. Riboflavin drops (Medio-Cross D®, Medio-Haus Medizinprodukte GmbH, Neudorf, Germany) were used in all cases, every 2 min. The imbibition time of the riboflavin was 20 min. Corneal cross-linking was performed using a CCL-365 Vario (MLase AG, Germering, Germany) with power of 9 mW/cm^2^ for 10 min, resulting in irradiance of 5.4 J/cm^2^. Riboflavin drops were continued every 2 min during the CXL treatment. A drop of topical Ketorolac 0.5% and 1 drop of Ofloxacin 0.3% were instilled and a bandage contact lens was applied at the end of the surgery. Postoperative treatment consisted of Ofloxacin 0.3% drops q.i.d. until contact lens removal, Dexamethasone 0.1% drops q.i.d., and artificial tear drops q.i.d.

### Postoperative assessment

Patients underwent routine postoperative assessment on the 1st, 3rd, 7th day and in the 1st, 3rd, 6th and 12th month. During the postoperative period from 1 month onwards, subjective refraction, slit-lamp biomicroscopy with grading of haze, intraocular pressure measurement and Precisio corneal topo/tomography were performed. Corneal haze was graded with an ordinal scale described by Fantes et al. [11]

### Sample size

The determination of the minimum sample size needed to obtain a valid estimation of the clinical data in terms of CDVA ratio is performed using the following formula based on the desired precision (d) and on a Gaussian assumption [12].

sample size $$ N={Z}^2\times \frac{\sigma^2}{d^2}\hbox{'} $$

where:

Z = 1.96, the standardized normal deviation corresponding to the 95% confidence level;

σ^2^ is the expected variance of the original data, which is estimated here below;

d is the desired precision of the deviation between the estimated value from the true value equal to 0.1.

The standard deviation can be estimated from the difference (h) between the highest and lowest values of the target parameter as σ = 0.25 × h for a symmetrical distribution shaped like an isosceles triangle.

The expected outcomes for the mean improvement in Corrected Distance Visual Acuity range from 1.0 to 1.6, equal to h = 0.6, where h is the highest value of the expected improvement in Corrected Distance Visual Acuity (1.6) minus the lowest value of the expected improvement in Corrected Distance Visual Acuity (1.0).

$$ \sigma =0.25\times h=0.25\times 0.6=0.15 $$

$$ N={1.96}^2\times {\left(0.25\times 0.6\right)}^2/{0.1}^2=8.64 $$

Considering an estimate of the follow up for the patients prudentially equal to 0.75, the number of cases to be enrolled for this study: N is equal to 8.64 ÷ 0.75 = 11.52 ≈ 12.

### Statistical analysis

D’Agostino-Pearson normality test was performed to assess the normality of the dataset (*p* > 0.05). A one-way analysis of variance (ANOVA) with a significant level p of 0.05 was used to determine differences between pre-operative, 3 months post-operative, 6 months post-operative and 12 months post-operative. Tukey’s multiple comparisons test was used to perform post hoc analysis. Statistically significant differences between the groups are indicated with (*) which represents a p-value < 0.05, (**) which represents a p-value < 0.01, and (***) which represents a p-value < 0.001.

## Results

24 eyes (13 right and 11 left eyes) of 24 patients meeting the inclusion criteria and signing the informed consent were included. There were 19 male and 5 female patients. Mean age was 28.92 years (SD 9.88, range 14 to 53). The average Max Ablation Depth (with epithelium) was 104.29 μm (SD 19.76, range 67.60 to 138.13). The average epithelium thickness is 50.92 μm (SD 3.39) and the average Max stromal ablation is 53.38 μm (SD 20.56). The preoperative epithelial profile maps were acquired using the Optovue OCT (Optovue, Fremont, CA, USA).

All the data presented a normal distribution (*p* > 0.05).

Mean preoperative spherical equivalent (SE) refractive error was − 0.74 D (SD 1.17, range − 3.35 to 0.82), while the cylinder error alone was − 3.06 D (SD 1.83, range − 0.50 to − 8.29). The minimum mean pachymetry value and Kmax values before the treatment were 483.93 μm (SD 33.90, range 405 to 569) and 51.38 D (SD 3.29, range 45.65 to 58.14), respectively.

At 3 months postoperatively, the SE was − 0.95 D (SD 2.31, range − 10.85 to 1.73), while the cylinder’s correction had a mean of − 1.19 D (SD 0.79, range − 2.80 to 0.82). In terms of pachymetry the post-operative measurements showed a mean thickness of 414.07 μm (SD 50.48, range 342 to 549) and the mean Kmax value dropped to 48.79 D (SD 2.98, range 43.99 to 55.45).

At 6 months postoperatively, the SE was − 0.97 D (SD 1.00, range − 4.59 to 0.25), while the cylinder’s correction had a mean of − 0.94 D (SD 0.56, range − 2.01 to 0.00). In terms of pachymetry, the post-operative measurements showed a mean thickness of 431.78 μm (SD 49.45, range 357 to 532) and the mean Kmax value dropped to 48.85 D (SD 2.58, range 43.84 to 53.60).

At 12 months postoperatively, the SE was − 1.05 D (SD 1.52, range − 5.25 to 0.40), while the cylinder’s correction had a mean of − 1.04 D (SD 0.80, range 0.0 to − 2.31). In terms of pachymetry, the post-operative measurements showed a mean thickness of 437.65 μm (SD 50.34, range 357 to 492) and the mean Kmax value dropped to 48.70 D (SD 2.58, range 44.02 to 53.04).

**Fig. 2:**
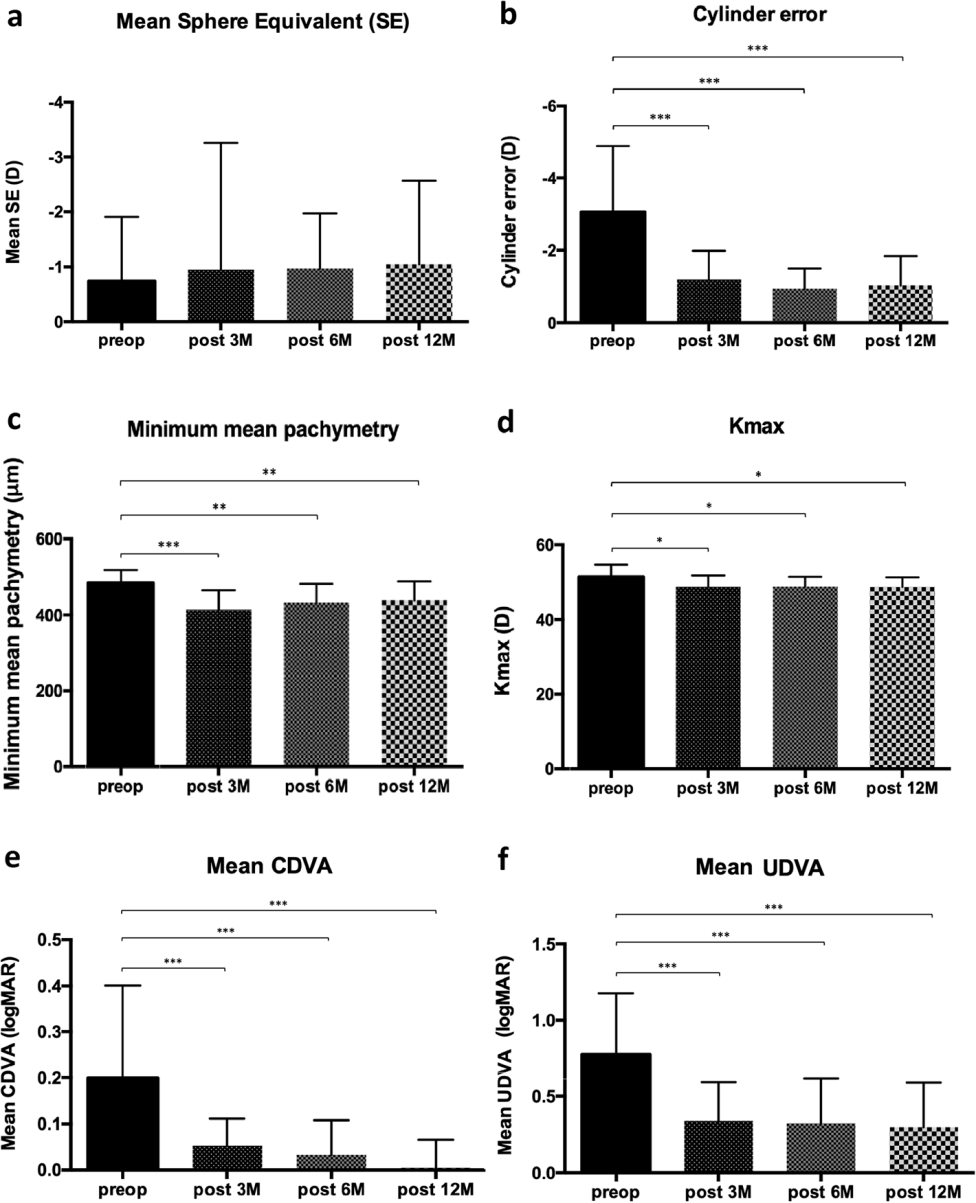
Pre and postoperative comparisons of the groups. (a) Mean sphere equivalent (SE); (b) Cylinder error; (c) Minimum mean pachymetry; (d) Kmax; (e) Mean corrected distance visual acuity (CDVA); (f) Mean uncorrected distance visual acuity (UDVA)

No statistically significant differences were detected among the groups for mean spherical equivalent (SE) refractive error (Fig. 2a). Comparing preoperative and postoperative data, a statistically significant reduction in terms of cylinder’s correction, minimum mean pachymetry, and Kmax were detected. Considering the postoperative data, no statistical differences were observed among the 3 M postop, 6 M postop and 12 M postop (Figs. 2b-d).

Mean preoperative CDVA (logMAR) was 0.20 (SD 0.20, range 0.00 to 0.70). Mean CDVA (logMAR) at 3 months was 0.05 (SD 0.05, range 0.00 to 0.20). Mean CDVA (logMAR) at 6 months was 0.03 (SD 0.07, range − 0.10 to 0.2). Mean CDVA (logMAR) at 12 months was 0.01 (SD 0.06, range − 0.10 to 0.10).

The mean preoperative UDVA (logMAR) was 0.80 (SD 0.40, range 0.10 to 1.3). The mean post-operative UDVA (logMAR) at 3 months was 0.34 (SD 0.25, range 0.00 to 1.00). The mean post-operative UDVA (logMAR) at 6 months was 0.32 (SD 0.29, range 0.00 to 1.30). The mean post-operative UDVA (logMAR) at 12 months was 0.30 (SD 0.29, range 0.00 to 1.00).

Comparing preoperative and postoperative data, a statistically significant reduction in terms of mean CDVA and mean UDVA was detected while no statistically significant differences were detected among the 3 M postoperative, 6 M postoperative and 12 M postoperative timepoints (Figs. 2e, f).

In terms of safety, no eye lost lines of best corrected acuity. 3 eyes gained no extra lines of visual acuity and the remaining 21 had a mean improvement of 3.21 lines (SD 1.68, range 0 to 6).

**Fig. 3:**
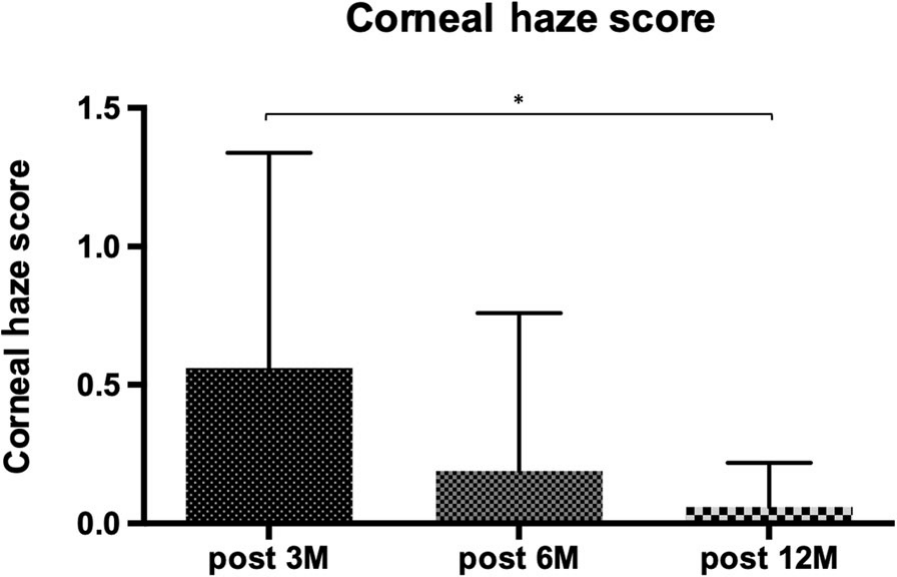
Corneal haze score comparison among the groups

The corneal haze was clinically graded according to the Fantes scale [11]. The mean haze score in the 3rd, 6th and 12th month was 0.56 (SD 0.78), 0.19 (SD 0.57) and 0.06 (SD 0.16), respectively. The detected mean haze score was statistically lower at 12 M postoperatively with respect to 3 M postoperatively showing that ultimately there was no opacity that could affect the patients’ visual acuity (Fig. 3).

**Fig. 4:**
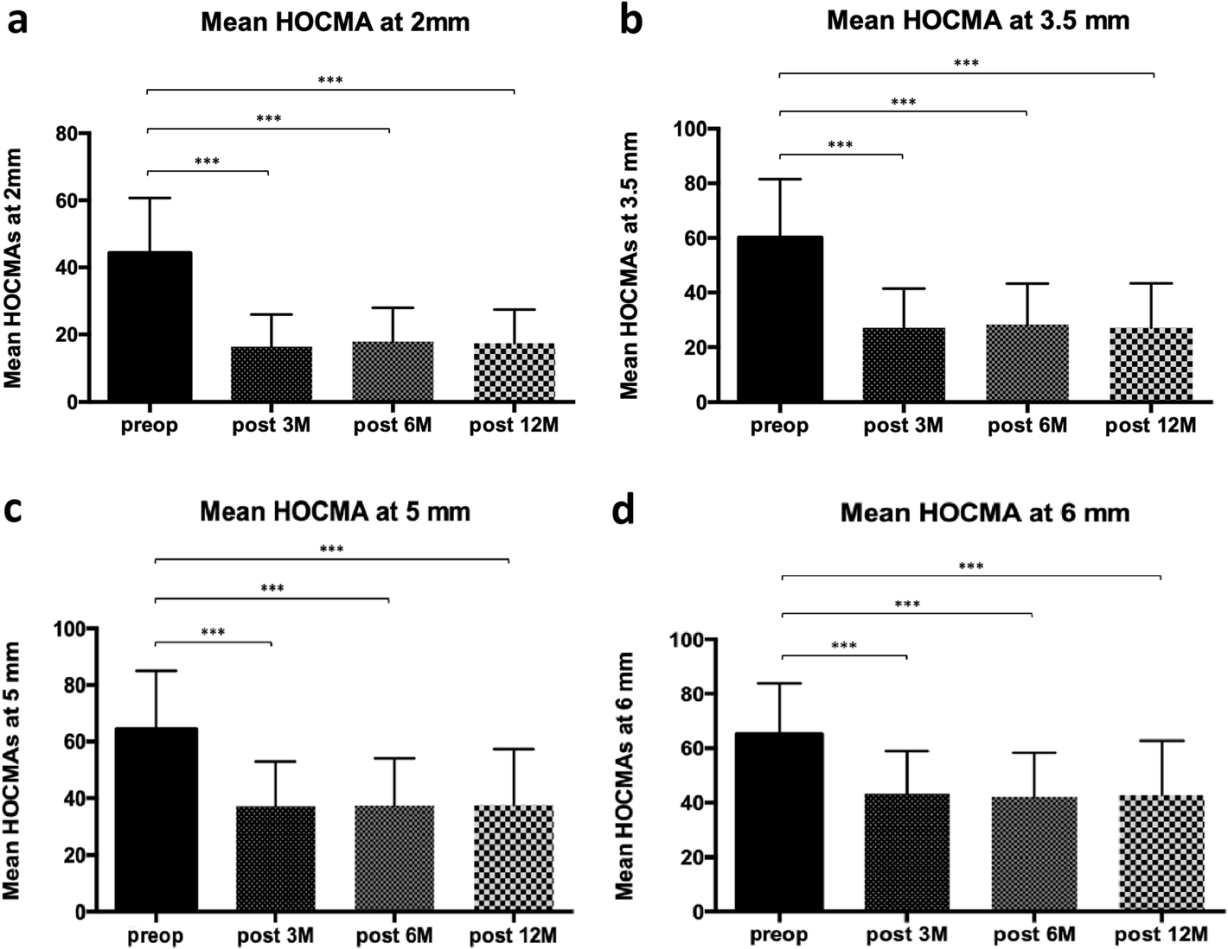
Mean higher order corneal morphological aberrations (HOCMAs) among the groups at different diameters. (a) HOCMAs at 2 mm; (b) HOCMAs at 3.5 mm; (c) HOCMAs at 5 mm; (d) HOCMAs at 6 mm

Considering the mean anterior HOCMAs analysis at 2 mm, a statistically significant reduction between preoperative and postoperative (3 M, 6 M and 12 M) was detected. In particular, the HOCMA at 2 mm were reduced by a factor of 3 after the surgery (Fig. 4a). The same trend was detected evaluating the mean anterior HOCMAs at 3.5 mm. In this case, the HOCMAs at 2 mm were reduced by a factor of 2 (Fig. 4b).

A similar trend was detected for mean anterior HOCMAs at 5 mm and 6 mm. Considering the mean anterior HOCMAs analysis at 5 mm, a statistically significant reduction by a factor of 2 between preoperative and postoperative (3 M, 6 M and 12 M) was detected (Fig. 4c). A similar trend was observed when we evaluated the mean anterior HOCMAs at 6 mm. In this case, the HOCMA at 2 mm were reduced by a factor of 3 (Fig. 4d).

## Discussion

This small series study assesses an approach that combines corneal customized transepithelial therapeutic ablation to treat the irregular corneal optics and accelerated cross-linking technique to strengthen the corneal tissue and stop the progression of keratoconus. Stiles–Crawford effect teaches that the most relevant portion of the cornea of interest for the distinct vision is the central 1.00 mm [13]. For this reason, a small central optical zone (1.0–1.5 mm) was selected to minimize corneal tissue ablation. In parallel to that, a customized connecting refractive zone with a constant radial slope and the continuity of the refractive power was planned with an aim to minimize the risk for glare and haloes as well as the risk for regression.

Different studies demonstrated that the combination of topography-guided photorefractive keratectomy (tPRK) and CXL procedures has the potential to improve both visual acuity and corneal stability [14, 15]. Kymionis et al. demonstrated the positive outcomes of combined tPRK with CXL in a noncomparative prospective cohort of patients with keratoconus and ectasia occurring after LASIK showing a significant improvement in UDVA, CDVA and stability [15]. Alessio et al. performed a prospective comparative study of tPRK with an excimer laser and CXL versus CXL alone and found higher improvement in visual acuity in the group of patients treated with combined customized treatment in comparison with CXL alone [16]. Kanellopoulos demonstrated the positive outcomes of the combination of surface ablation with CXL in a single case report of a patient treated with CXL followed by a tPRK procedure after 1 year [17]. In addition, Kanellopoulos et al. published a retrospective comparison of same-day simultaneous collagen CXL and tPRK versus CXL sequentially followed by tPRK after 6 months for treatment of keratoconus. They reported a better average improvement in UDVA in the same-day group with respect to the sequential group [18]. Kontadakis et al. prospectively compared the topographic and refractive results in patients treated with simultaneous tPRK and CXL with those patients treated with CXL alone demonstrating that the combined technique improved the vision of the treated patients in comparison with CXL alone while similar results in terms of postoperative stability were detected [14].

Epithelial removal by use of the concept of transepithelial PTK was chosen to take advantage of the smoothening effect that the epithelial remodeling has on the underlying corneal stroma. Due to the uneven thickness of the epithelium covering the irregular stromal surface in keratoconus, the predefined ablation profile will in addition to the epithelium, ablate the parts of the protruding stroma, where the epithelial thickness is below the preset ablation depth. This approach has been widely reported to be successful and advantageous to the mechanical or chemical epithelial removal [19, 20]. The current approach went a step further by regularizing the corneal surface beyond the effect of the smoothening effect of the epithelial remodeling, while keeping the low stromal ablation depth and volume. Good results in treatment of keratoconus with the current system using topography-guided ablation with larger optical zones and combined with CXL has been shown earlier [7], but the stromal ablation depth and volume were necessarily higher with that approach [7]. In order to further decrease the stromal tissue consumption, preoperative epithelial mapping may be used. Knowing that the epithelial thickness is generally lower in keratoconus than in normal virgin eyes [21], using the real thickness instead of the manufacturer’s proprietary algorithm in the programming of the treatment could spare a certain additional amount of stromal tissue. In addition, the results obtained in this study are comparable with the ones presented by Kontadakis et al. [14] and Alessio et al. [16] in terms of mean UDVA and mean CDVA demonstrating that the combined CCR and CXL technique proposed in this study can offer a considerable and effective improvement for keratoconic patients.

## Conclusions

In conclusion, we believe that CCR combined with CXL offers promising results and is an effective treatment in keratoconic patients with contact lens intolerance.

## Data Availability

The datasets used and/or analyzed during the current study are available from the corresponding author on reasonable request.
